# A Classification Method Based on Principal Components of SELDI Spectra to Diagnose of Lung Adenocarcinoma

**DOI:** 10.1371/journal.pone.0034457

**Published:** 2012-03-26

**Authors:** Qiang Lin, Qianqian Peng, Feng Yao, Xu-Feng Pan, Li-Wen Xiong, Yi Wang, Jun-Feng Geng, Jiu-Xian Feng, Bao-Hui Han, Guo-Liang Bao, Yu Yang, Xiaotian Wang, Li Jin, Wensheng Guo, Jiu-Cun Wang

**Affiliations:** 1 Department of Thoracic Surgery, Shanghai Chest Hospital, Shanghai Jiao Tong University, Shanghai, China; 2 Ministry of Education Key Laboratory of Contemporary Anthropology and State Key Laboratory of Genetic Engineering, School of Life Sciences and Institutes of Biomedical Sciences, Fudan University, Shanghai, China; 3 Department of Pulmonary Medicine, Shanghai Chest Hospital, Shanghai Jiao Tong University, Shanghai, China; 4 Shanghai Chest Cancer Research Institute, Shanghai Chest Hospital, Shanghai Jiao Tong University, Shanghai, China; 5 Department of Biostatistics and Epidemiology, University of Pennsylvania, Philadelphia, Pennsylvania, United States of America; Queen Elizabeth Hospital, Hong Kong

## Abstract

**Purpose:**

Lung cancer is the leading cause of cancer death worldwide, but techniques for effective early diagnosis are still lacking. Proteomics technology has been applied extensively to the study of the proteins involved in carcinogenesis. In this paper, a classification method was developed based on principal components of surface-enhanced laser desorption/ionization (SELDI) spectral data. This method was applied to SELDI spectral data from 71 lung adenocarcinoma patients and 24 healthy individuals. Unlike other peak-selection-based methods, this method takes each spectrum as a unity. The aim of this paper was to demonstrate that this unity-based classification method is more robust and powerful as a method of diagnosis than peak-selection-based methods.

**Results:**

The results showed that this classification method, which is based on principal components, has outstanding performance with respect to distinguishing lung adenocarcinoma patients from normal individuals. Through leaving-one-out, 19-fold, 5-fold and 2-fold cross-validation studies, we found that this classification method based on principal components completely outperforms peak-selection-based methods, such as decision tree, classification and regression tree, support vector machine, and linear discriminant analysis.

**Conclusions and Clinical Relevance:**

The classification method based on principal components of SELDI spectral data is a robust and powerful means of diagnosing lung adenocarcinoma. We assert that the high efficiency of this classification method renders it feasible for large-scale clinical use.

## Introduction

Lung cancer is the leading cause of cancer death worldwide, and it ranked second among new cancer cases in the United States in 2009 [1]. In China, the incidence of lung cancer, 35 cases per 100,000 people per year, makes it the most common form of cancer in the country. Over 20% of cancer deaths in China are caused by lung cancer [2]. For this reason, the Ministry of Health of China has listed lung cancer as the most important item on its cancer prevention and control agenda [2]. Lung cancer can be categorized into small cell lung cancer and non-small cell lung cancer (NSCLC) according to histological criteria. NSCLC accounts for about 85% of all cases of lung cancer and is further categorized into the specific sub-types: adenocarcinoma, squamous cell carcinoma, and large cell carcinoma [3]. Due to the lack of effective techniques for early diagnosis, most patients are at an advanced stage when diagnosed, leading to the poor outcomes. The 5-year survival rate is only about 10–15% for NSCLC [4], [5].

A great deal of effort has been invested in the identification of markers for the screening of malignancies during early diagnosis. For example, proteomics technology has been applied extensively to the study of proteins involved in carcinogenesis [6], [7]. The latest development in systematic analysis of protein composition in cells (i.e., protein profiling) has shown that protein profiles are closely aligned with cellular activities. Proteomics technology may be a promising tool in cancer screening and diagnosis [8]. In contrast, other high-throughput methods, such as transcriptome profiling of mRNA and miRNA, have shown only limited power in reflecting tumor heterogeneity [5], [9], [10]. In addition, protein profiling is also highly versatile. It can be applied to different kinds of samples including tissues and body fluids [8], [10], [11].

Surface-enhanced laser desorption/ionization time-of-flight mass spectrometry (SELDI-TOF-MS, SELDI), a high-throughput protein profiling method, has been used successfully to distinguish cancer from non-cancer and normal controls [9], [12]. The existing methods for analyzing SELDI spectral data include two steps, peak screening and data analysis [13], [14]. The aim of peak screening is to identify high-quality peaks (signal-to-noise ratio >2) through baseline subtraction, normalization, peak detection, and peak alignment [13]. Then the data analysis was aimed to detect significant peaks, which could be taken as biomarkers in corresponding disease studies [15]. In case-control studies, peaks that are significantly different between cases and controls are detected using statistical analysis (ttest, ANOVA), or data-mining based methods, such as the classification and regression tree model (CART), the decision tree method (DT), the support vector machine (SVM), and the linear discriminant approach (LDA) [16], [17]. SELDI has been applied widely in the screening of biomarkers in prostate, pancreatic, gastric, breast, nasopharyngeal, liver, ovarian, thyroid and lung cancers [9], [10], [12], [18], [19]. For lung cancer screening, it has been shown to be more powerful than the more common serum markers, such as Cyfra21-1 and NSE [20], [21]. Its utility for diagnosis and prediction of prognosis in non-smoking patients has also been reported [22]. These peak-selection-based methods have several limitations. First, peak-selection-based methods focus on high peaks, which represent high concentrations of proteins. However, the selected peaks may be common among cases and controls and may not indicate any difference between these two groups. Smaller peaks that differ between groups, however, may have more predictive power. These smaller peaks can be ignored during the peak screening step. Second, peak-selection-based methods take peaks as independent and ignore the information inherent in their locations. It is believed that the combination of the peaks and relationship among their relative locations may contain information that is useful in group discrimination. Third, the results of peak-selection-based methods can vary from sample to sample. Often the tumor markers established in one study are usually poorly validated subsequent studies, and the sensitivity and specificity of diagnostic and prediction models are usually not well reproduced, even within the same lab [23].

In light of these limitations, we constructed a classification method for diagnosis or prediction of diseases based on principal components of SELDI spectral data without peak selection. The classification method includes two optimization steps, first screens candidate principal components, and second hunts the optimized model. To evaluate the performance of this classification method based on principal components of SELDI spectral data, we compared it to the peak-selection based methods DT, CART, SVM, and LDA through leaving-one-out, 19-fold, 5-fold, and 2-fold cross validation.

## Materials and Methods

### Patients and samples

Plasma samples from 71 lung adenocarcinoma patients were collected from patients who underwent pulmonary resection for primary lung cancer at Shanghai Chest Hospital. All participants provided informed consent. The diagnosis and histological classification of the tumors were carried out following the criteria from AJCC (American Joint Committee on Cancer) [24]. The demographic and clinical characteristics of the subjects are summarized in Table 1. No patients received radiotherapy or chemotherapy prior to surgery. Twenty-four normal samples were collected from healthy volunteers who took physical examinations at the same hospital. The research was conducted with the official written approval (written form) of the Biomedical Ethics Committee of Fudan University, Shanghai, China.

**Table 1:** Features of lung adenocarcinoma patients.

Pathological parameters	Tumors^a^	Sex		Age(y)
		Male	Female	
**Tumor Size**				
**T_1_**	5 (7.04%)	5	0	38–64
**T_2_**	46 (64.79%)	25	21	41–72
**T_3_**	10 (14.08%)	4	6	40–75
**T_4_**	10 (14.08%)	3	7	37–75
**Nodal involvement**				
**N_0_**	25 (35.21%)	12	13	38–75
**N_1_**	25 (35.21%)	14	11	51–72
**N_2_**	21 (29.58%)	11	10	37–69
**Metastasis**				
**M0**	70 (98.59%)	36	34	37–75
**M1**	1 (1.41%)	1	0	42
**AJCC Stage**				
I	17 (23.94%)	11	6	38–68
II	26 (36.62%)	13	13	49–75
III**A**	18 (25.35%)	10	8	40–72
III**B**	9 (12.68%)	2	7	37–75
IV	1 (1.41%)	1	0	42
**Histologic grade**				
**Poor**	28 (39.44%)	20	8	37–75
**Moderate**	32 (45.07%)	13	19	38–75
**Good**	11 (15.49%)	4	7	4172

a Number of cases. The numbers in the parenthesis stand for the percentage.

### SELDI-TOF-MS

Three-microliter plasma samples were diluted with 2-fold buffer U9 (9 M urea, 2% CHAPS, 50 mM Tris-Hcl, 1% DTT, pH 9.0) and shaken on ice for 30 min. Then 108 µL binding buffer (100 mM NaAc, pH 4) was added to the plasma which made a final dilution of 39-fold. The SELDI ProteinChip arrays of weak cation exchange (WCX-2) from Ciphergen Biosystems were used for protein capture. Chips were put into a bioprocessor and washed twice with 200 µL of binding buffer (50 mM NaAc, pH 4) for each well with gentle shaking for 5 min, keeping the surface of each spot wet. Then 100 µL of the diluted plasma was added to each well and shaken at 4°C for 1 hour. The wells were washed twice with 200 µL of binding buffer, followed by washing with HPLC water. They were then allowed to dry. Then 0.5 µL of sinapinic acid was applied to each spot twice. The arrays were allowed to air-dry and then subjected to SELDI analysis and, read using a Protein-Chip reader. Seven peptides, including 1084.247-[Arg8]-vasopressin, 1637.903-somatostatin, 2147.500-dynorphin A, 2933.500-ACTH[1–24]human, 3495.941-insulin B-chain (bovine), 5807.653-[Arg]-Insulin, and 7033.614-hirudin BKHV, were randomly selected from an all in-one peptide standard (NP20 chip, Ciphergen Biosystems) to calibrate the PBS-II-c ProteinChip reader (Ciphergen Biosystems). Each spot was scanned with a laser intensity of 185 and a detector sensitivity of 8 to acquire an optimal mass of 1 to 30 kDa and a maximum mass of 50 kDa. To evaluate the reliability and stability of our assay, data from one plasma sample in 6 randomized chip locations was analyzed.

### Data preprocessing

Suppose we have 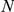 individuals assayed by SELDI. Each spectrum is characterized as 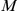 signal intensities at corresponding M/Z locations. We denote the signal intensity of the 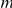 M/Z point of individual 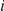 as *S_im_*. In our dataset, all individuals share the same M/Z locations, so the entire dataset can be stored in an 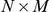 signal intensity matrix. The following procedures are applied to normalize multiple SELDI spectra:

1. We extract raw signal intensity values at each M/Z location using Ciphergen's ProteinChip® software. No additional processing option (background subtraction or normalization) is employed.
2. We perform logarithm transformation of all raw intensity values to approximately stabilize the signal variance while retaining the biological interpretation.
3. For each individual, we estimate the intensity background of each M/Z location with the median value of a sliding window. Each sliding window is centered at the M/Z location of interest and spans 24 M/Z points in both directions. The median statistics are chosen for their known robustness (24) against outliers (sharp peaks).
4. We performed intensity background subtraction at each M/Z point. The subtraction is performed on logarithm scale which stands for the log-ratio of signal against background, thus the result is still biological meaningful.
5. We perform multiple spectrum quantile normalization. Quantile normalization is used to minimize the bias across different spectra, in which the intensity distribution of every spectrum is forced to equal that of the others.

### Classification method based on principal components of SELDI spectral data

The procedures of the classification method based on principal components of SELDI spectral data are as follows:

**Step 1: Based on preprocessed data, principal component analysis (PCA) is applied to the SELDI spectral data to obtain orthogonal linear combinations of the SELDI spectra.**

**Step 2: Candidate principal components are selected based on group difference.**

**Step 3: The selected principal components are jointly incorporated into logistic regression model and, based on certain criteria, the optimal classification model based on principal components of SELDI spectral data is obtained.**

In **Step 2**, principal components which are significantly different between cases and controls (significance level 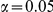) are selected in the classification method based on principal components of SELDI spectral data. This is different from general principles of screening principal components, eigenvalues greater than 1 or contribution larger than 80 percent). In **Step 3**, the selected principal components are jointly incorporated in logistic regression model based on three criteria for relative logistic regression models, R square, the Hosmer-Lemeshow statistic (which indicates the goodness-of-fit of the classification model), and accuracy from leaving-one-out cross validation. Then the optimal classification model based on principal components of SELDI spectral data is found. The accuracy of leaving-one-out cross validation is considered more important than R square or the Hosmer-Lemeshow statistic when the two criteria are within a certain range.

### Comparison to peak-selection-based methods

We then compared the performance of the classification method based on principal components of SELDI spectral data here developed to the peak-selection-based methods DT, CART, SVM and LDA. We used leaving-one-out, 19-fold, 5-fold, and 2-fold cross validation. The construction and cross-validation of the classification model based on principal components of SELDI spectral data is demonstrated above.

The peak screening processes of SELDI spectral data and data analysis for peak-selection-based methods DT, CART, SVM, and LDA are demonstrated below:

**Step 1: Peak screening process.** After baseline subtraction and normalization, peak detection was used to eliminate any peaks whose intensities were below a specified signal-to-noise (S/N) threshold guided, for example, by the magnitude of the signal-to-noise ratio (SNR, in this paper, SNR = 2). Then peak alignment was applied by generating an interval around each peak centered at the m/z value for the peak (0.3%). Then the maximum value was taken as the height of peak [13].

**Step 2: Data analysis and cross-validation.** After screening, the data from the selected peaks was entered into the Tanagra software package, and analysis and cross-validation of DT, CART, SVM and LDA was performed.

## Results

### Reproducibility

We evaluated the reliability and stability of the technique by analyzing data from one plasma sample in 6 randomized locations on chips. The coefficient variations of M/Z values and protein intensity of randomly selected proteins were <1‰ (*P* = 4.88E-04) and 0.12 (*P*<0.2), respectively, confirming that SELDI-TOF-MS offers a stable and reliable measurement.

### Application of the classification method based on principal components of SELDI spectral data

We applied the classification method based on principal components developed in this paper to SELDI spectral data from 71 lung adenocarcinoma patients and 24 healthy controls. First, the first seven principal components were considered in the candidate alignment. Of these, the seventh principal component was at the edge of the contribution curve cut-off [25], [26]. Then the logistic regression model was applied to each principal component to assess its association with group status. The results showed that the first principal component (PC1), sixth principal component (PC6), and seventh principal component (PC7) were significantly different in different groups, with *P*<0.01, *P* = 0.03 and *P* = 0.03, respectively (Table 2). PC1 was the most significant one, accounting for 53.9% of the difference between two groups, while PC6 and PC7 accounted for 5% and 6%, respectively. Then PC1, PC6, and PC7 were jointly incorporated in logistic regression models to construct a classification model (Table 3) in which PC1 was included in all the models. All the potential classification models had conceivable indices of goodness-of-fit (Hosmer-Lemeshow statistic) [27]. Cross-validation results showed that the logistic classification model based on PC1 and PC7 performed as well as that on PC1, PC6, and PC7, with the same values of accuracy (Table 3). The former was preferred because it was equally efficient but more concise. The optimal classification model based on principal components (that logistic classification model based on PC1 and PC7) was found to account for 61.71% of the difference between two groups.

**Table 2:** Candidate principal components.

PC	PC1	PC2	PC3	PC4	PC5	PC6	PC7
**Proportion (%)** a	18.1	10.5	7.69	4.90	4.41	3.43	3.16
***P*** ** value** b	<0.01*	0.39	0.12	0.48	0.48	0.03*	0.03*
**R square** c	0.54	0.01	0.03	0.01	0.01	0.05	0.06

a Contribution of each PC to the whole variation.

b *P* value of the coefficient testing of logistic regression analysis on each PC.

c Fitness index of each logistic regression model on single PC.

**Table 3:** Summary of classification models based on principal components of SELDI spectral data.

Model	R square	Hosmer-Lemeshow statistic	Cross validation accuracy
pc1	0.5338	3.01 (0.93)	92.63%
pc1 pc6	0.5591	1.32 (1.00)	92.63%
***pc1 pc7***	***0.6171***	***0.99 (1.00)***	***95.79%***
pc1 pc6 pc7	0.6330	0.33 (1.00)	95.79%

**Figure 1:**
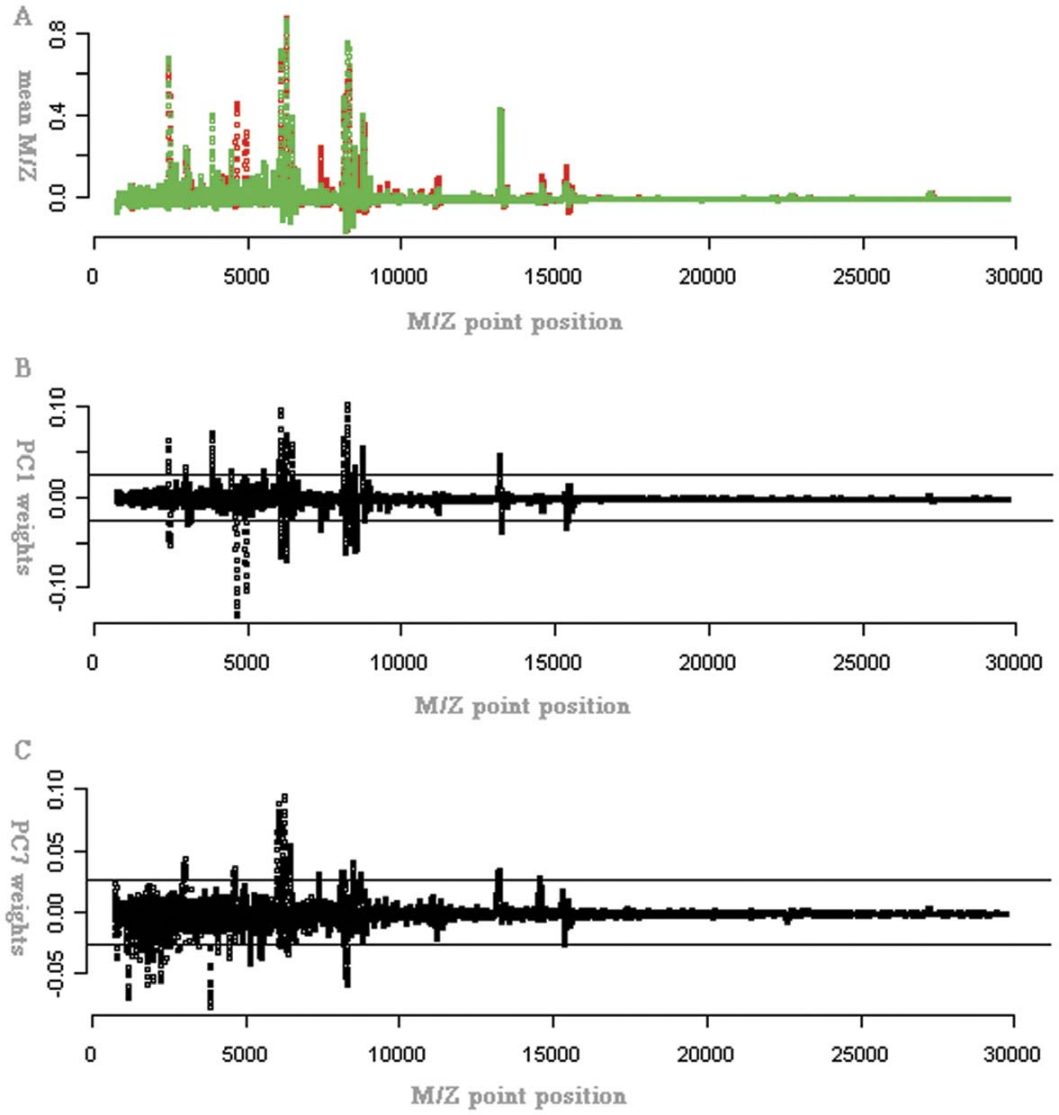
M/Z means of cases and controls and the weights of PC1 and PC7 on the spectrum. **A**) The M/Z means of cases (red) and normal controls (green) at each M/Z point. **B**) The weights of PC1 at each M/Z point. **C**) Weights of PC7 at each M/Z point. Horizontal lines in Figure 1B and 1C represent 3*SD of corresponding PC on the spectrum. The data used here are the normalized SELDI data obtained from 71 lung adenocarcinoma patients and 24 normal individuals.

**Figure 2:**
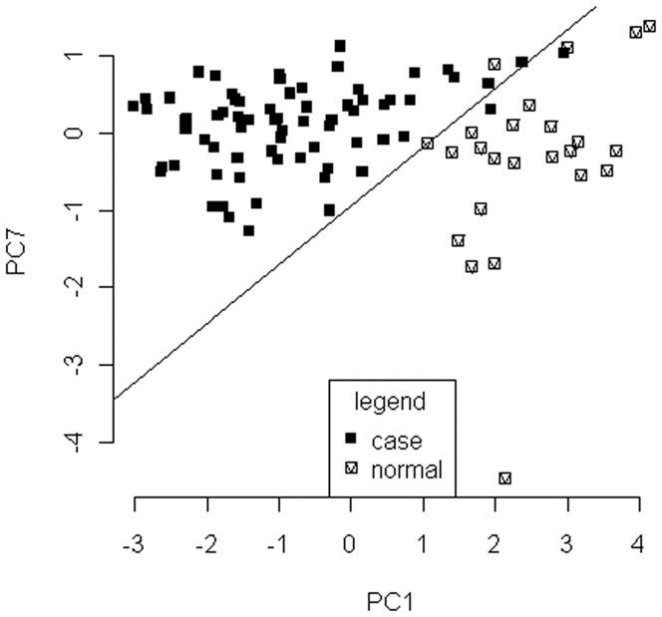
Classification method based on principal components of SELDI spectral data and experimental data. Two cases and two normal individuals had been misclassified into opposite groups. The black squares indicate case individuals, and white squares with “V” shapes in the middle represent normal individuals. The data used here are the normalized SELDI data obtained from 71 lung adenocarcinoma patients and 24 normal individuals.

The explicit formulation of the optimal classification model based on principal components of SELDI spectral data is shown in Table 4. The relationship between principal components and SELDI spectral data was important for selecting key M/Z points contributing greatly to each PC. In particular, we displayed the mean M/Z value at each point for cases and controls (Figure 1A). The weights of PC1 and PC7 on the M/Z points are presented in Figure 1 (Figure 1B for PC1 and Figure 1C for PC7). In Figures 1B and 1C, the horizontal lines represent +/−3*SD of corresponding principal component weights on all M/Z points. In Figures 1B and 1C, the weights beyond the two horizontal lines indicate that the corresponding M/Z points contributed more than other M/Z points to the related principal component. Interestingly, Figure 1 shows that not only maximum peaks (the two M/Z points between 5,000 and 10,000) and significant peaks (M/Z points around 5,000) contributed to classification model based on principal components of SELDI spectral data but also that those peaks were not very high (M/Z points near zero). Figure 2 shows the result of the optimal classification model based on principal components of the SELDI spectral data, in which 2 cases and 2 normal observations were misclassified.

**Table 4:** Optimal classification model based on principal components of SELDI spectral data.

Parameter	Coef (STDErr)	OR (95%CL)	*P* value
**Intercept**	−5.49(1.92)		<0.01
**PC1**	4.05(1.40)	57.22(3.72, 881.07)	<0.01
**PC7**	−5.30(2.27)	0.005(<0.001, 0.43)	0.02

Coef, coefficient.

STDErr, standard error.

OR, odds ratio.

CL, confidence level.

### Comparison with peak-selection based methods

**Figure 3:**
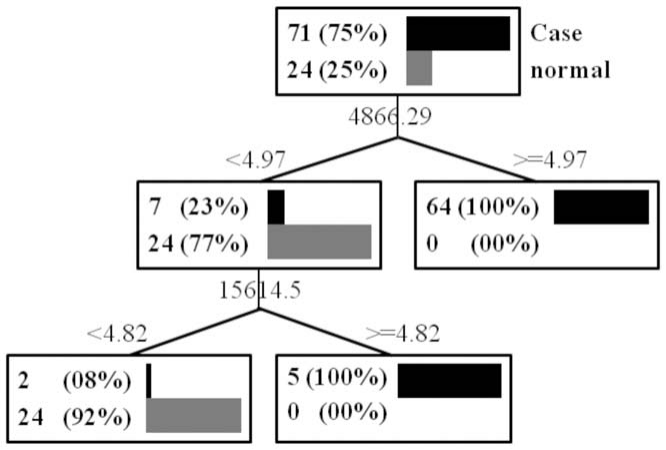
Decision-tree-based classification model and experimental data. Two peaks that identified using a decision-tree-based classification model are shown, with 2 cases misclassified into control groups. The data used here are the peaks selected through baseline subtraction, normalization, peak detection, and peak alignment of SELDI data obtained from 71 lung adenocarcinoma patients and 24 normal individuals.

The construction and cross-validation of DT, SVM, LDA, and CART were performed using Tanagra software. The criteria used for DT was that confidence level 0.25 and minimum size of leaves 5. The classification result of DT on 71 lung adenocarcinoma and 24 controls was shown in Figure 3. The kernel used in SVM was a polynome with a polynome exponent of 1. The criteria used for CART were minimum node size to split 10 and a pruning set size of 15%.

Cross-validation results for DT, SVM, LDA, CART, and the classification method based on principal components of SELDI spectral data are shown in Table 5. The classification method based on principal components here developed completely outperformed peak-selection-based methods DT, SVM, LDA and CART with respect to sensitivity, specificity, and accuracy as determined by leaving-one-out, 2-fold, 5-fold, and 19-fold cross validation. Cross-validation also showed that the performance of the classification model based on principal components was similar across the leaving-one-out, 19-fold, 5-fold, and 2-fold cross validation, which indicated that it was not sensitive to sample size.

**Table 5:** Cross-validation results of DT, SVM, LDA, CART, and our method.

Cross-validation	DT	SVM	LDA	CART	Our method
**Leaving-one-out** a					
	91.55%	95.77%	88.73%	90.14%	97.18%
	87.50%	83.33%	91.67%	70.83%	91.67%
	90.53%	92.63%	89.47%	85.26%	95.79%
**19-fold**					
	91.55%	94.37%	90.14%	92.96%	97.18%
	87.50%	83.33%	87.50%	79.17%	91.67%
	90.53%	91.58%	89.47%	89.47%	95.79%
**5-fold**					
	94.37%	94.37%	85.92%	90.14%	97.18%
	79.17%	79.17%	87.50%	58.33%	91.67%
	90.53%	90.53%	86.32%	82.11%	95.79%
**2-flod**					
	94.37%	94.37%	77.46%	94.37%	95.77%
	62.50%	58.33%	75.00%	54.17%	91.67%
	86.32%	85.26%	76.84%	84.21%	94.74%

DT, decision-tree-based classification model; SVM, support vector machine; LDA, linear discriminant approach; CART, classification and regression tree.

a The first line is the true positive rate (sensitivity); the second line is the true negative rate (specificity); and the third line is accuracy.

## Discussion

In this paper, we propose a classification method based on principal components of SELDI spectral data. Principal component analysis is mathematically defined as an orthogonal linear transformation that transforms the data into a new coordinate system such that the greatest variance by any projection of the data comes to lie on the first coordinate (called the first principal component), the second greatest variance on the second coordinate, and so on. Principal component analysis has already been applied in SELDI data analysis. In 2001, Nilsen et al. used principal component analysis to select peaks to visually identify natural clusters [28]. In 2003, Lilien et al. used principal component analysis as a dimension-reduction method [29]. Kernel principal component analysis combined with a logistic regression model has been applied to the study of gene expression [30]. However, most of the current methods with PCA analysis is first using PCA to find the first couple principal components, and then using the first couple components in a second step classification. The first two principal components explain the biggest variability in the spectra. However, they can be the same in cases and controls, and may not be the most predictive of the group status. In our method, PCA is applied to obtain orthogonal linear combinations of SELDI spectral data. Since the final goal is to distinguish cases from controls, the most important information is the principal components that are different between groups, not the ones that explain the most variability. We are the first to emphasize selection of the principal components that are most predictive of the group status. This is a simple yet important idea.

Unlike peak-selection-based methods, the classification method based on principal components of SELDI spectral data took each SELDI spectrum as a whole entity, and then PCA was applied to the SELDI spectral data to obtain orthogonal linear combinations of the SELDI spectra. The candidate principal components are selected just based on group differences. Then the selected principal components are jointly incorporated into logistic regression model and, based on certain criteria, the optimal classification model based on principal components of SELDI spectral data is obtained. The classification method based on principal components of SELDI spectral data presents several advantages. First, candidate principal components are selected based on group differences, so principal components that account for less variability among the data but possess more power for group discrimination can be selected. This would ordinarily include the PC2, which in our data was not included in the classification model because it was not significantly different across cases and controls. PC7 was included in the classification model because it showed significant differences between groups while accounting for only moderate variance among the SELDI spectra data. We only considered the first seven principal components in this paper, though other principal components, which were smaller than PC7, could also be detected based on group differences if higher power had been desired. In contrast, these smaller principal components may be ignored in other principal-component-based methods. Second, the classification model based on principal components of SELDI spectral data takes into account the pattern of the spectra, such as the combination and the relative locations of the M/Z values, while the peak-selection-based methods take peaks as independent entities. The principal components are linear combinations of the whole spectra which directly represents the differences in patterns of spectra from cases to controls. In Figures 1B and 1C, the weights beyond the two horizontal lines indicate that the corresponding M/Z points contribute more than other M/Z points to the related principal component. Interestingly, Figure 1 shows that not only maximum peaks (the two M/Z points between 5,000 and 10,000) and peaks that are significantly different between groups (M/Z points around 5,000) contributed to the classification model based on principal components of SELDI spectral data but also that smaller peaks (M/Z points near zero) provided informative probability that was used to distinguish cases from controls along with spectra. Lastly and most important, the reproducibility of results from the classification method based on principal components of SELDI spectral data was found to be better than that of peak-selection-based methods. As shown in Table 5, the results of leaving-one-out, 19-fold, 5-fold, and 2-fold cross-validation showed that the sensitivity, specificity, and accuracy of our method completely outclassed those of peak-selection-based methods (DT, CART, SVM, and LDA).

Protein profiling using two-dimensional electrophoresis, MALDI, SELDI, and other methods has been used for diagnosis, classification, prognosis, and drug discovery in the study and clinical treatment of numerous cancers [7], [8], [9], [10], [12], [31], [32], [33]. The samples used in protein profiling have included tissues, exhaled breath condensate, blood, and others available specimens. Blood samples, which are minimally invasive and readily available, are the specimens of choice for cancer screening and early diagnosis. Successful implementation of screening in several cancers has led to reduced mortality and improved outcomes [10]. Although most lung cancer patients are at advanced stages when their condition is diagnosed, the 5-year survival rate can increase to 52% if they are diagnosed in stage I and resected at once [34]. This is why it is a top priority to screen lung cancer and diagnose it as early as possible. Based on these reports, in the present study, plasma samples from lung adenocarcinoma patients and normal controls were analyzed using the well-established protein profiling method SELDI-TOF-MS to explore the possibility and accuracy of lung cancer screening and early diagnosis. The classification method based on principal components of SELDI spectral data could be applied to other types of spectral data, such as that collected from other types of cancer or other tissue or fluid samples when available.

There are several reported disadvantages of SELDI-TOF-MS [15], [35]. Tumor markers that seem very accurate in one study may have middling results in others. The same is true of marker sensitivity and specificity, even within the same lab [23]. Many possible reasons for this phenomenon have been suggested. The most important possible cause of this disadvantage is the fact that conventional peak-calling methods cannot promise full power in discriminating cases from controls. Although a typical SELDI-TOF-MS profile has up to 15,500 data points representing between 500 and 20,000 M/Z values, many studies call for fewer than 100 peaks in a SELDI spectrum analyses using software [8], [20], [21], [23]. This is less than what can be scored manually. We observed that some peaks with moderate M/Z values and some plateaus with high M/Z values were not identified by the software. Sometimes moderate peaks and plateaus bare differentially expressed between cases and controls. This makes them valuable as biomarkers, indicating the disease. For example, when using the conventional calling method with Biomarker Wizard software, only 21 of the M/Z peaks were detected as different across the case group and the control group. Another disadvantage of SELDI-TOF-MS is that the proteins cannot be identified directly. However, as mentioned above, that does not affect the accuracy of diagnosis of lung adenocarcinoma. With the help of appropriate methods of statistical analysis, cancer can be identified correctly by SELDI-TOF-MS profiling. From there, diagnosis based on proteomic signatures can be expected as a complement to routine measurement, such as X-rays, CTs, and MRI.

Generally speaking, the classification method based on principal components of SELDI spectral data developed in this paper is a robust and powerful method for diagnosis of lung adenocarcinoma. It may become a valuable part of the toolbox of cluster and discriminatory analysis. We propose that the high efficiency of the classification model based on principal components of SELDI spectral data renders it feasible for the large-scale clinical diagnosis of lung adenocarcinoma.
